# Supplementary material for: Discordance of HER2-Low between Primary Tumors and Matched Distant Metastases in Breast Cancer
Source: Cancers (Basel). 2023 Feb 23;15(5):1413. doi: 10.3390/cancers15051413 (PMC10000561; doi:10.3390/cancers15051413)
Supplement: Supplementary file 1 [file cancers-15-01413-s001.zip › Supplement/Table S1.docx]

**Table S1:** Change of HER2 status between primary tumor and metastasis in the HER2-negative cohort (n=127)

|  |  | **Metastasis** | | |
| --- | --- | --- | --- | --- |
| **Primary tumor** |  | **HER2-zero**  **(n=31, 24.4%)** | **HER2-low**  **(n=86, 73.5%)** | **HER2 positive**  **(n=31, 24.4%)** |
|  | **HER2-zero**  **(n=49, 38.6%)** | 13 (10.2%) | 34 (26.8%) | 2 (1.6%) |
|  | **HER2-low**  **(n=78, 61.4%)** | 18 (14.2%) | 51 (40.2%) | 9 (7.1%) |
