# Supplementary material for: Discordance of HER2-Low between Primary Tumors and Matched Distant Metastases in Breast Cancer
Source: Cancers (Basel). 2023 Feb 23;15(5):1413. doi: 10.3390/cancers15051413 (PMC10000561; doi:10.3390/cancers15051413)
Supplement: Supplementary file 1 [file cancers-15-01413-s001.zip › Supplement/Table S2.docx]

**Table S2:** Change of HER2 status between primary tumor and metastasis in the entire cohort (n=148)

|  |  | **Metastasis** | | |
| --- | --- | --- | --- | --- |
| **Primary tumor** |  | **HER2-zero**  **(n=31, 20.9%)** | **HER2-low**  **(n=86, 58.1%)** | **HER2 positive**  **(n=31, 20.9%)** |
|  | **HER2-zero**  **(n=49, 33.1%)** | 13 (8.8%) | 34 (23.0%) | 2 (1.4%) |
|  | **HER2-low**  **(n=78, 52.7%)** | 18 (12.2%) | 51 (34.5%) | 9 (6.1%) |
|  | **HER2 positive**  **(n=21, 14.2%)** | 0 | 1 (0.7%) | 20 (13.5%) |
