# Supplementary material for: Discordance of HER2-Low between Primary Tumors and Matched Distant Metastases in Breast Cancer
Source: Cancers (Basel). 2023 Feb 23;15(5):1413. doi: 10.3390/cancers15051413 (PMC10000561; doi:10.3390/cancers15051413)
Supplement: Supplementary file 1 [file cancers-15-01413-s001.zip › Supplement/Table S3.docx]

**Table S3:** Change of HER2 status in different metastatic sites in the HER2-negative cohort (n=127)

|  | **No change** | **HER2 change** | | | | |  |
| --- | --- | --- | --- | --- | --- | --- | --- |
|  |  | **Total** | **HER2-zero to HER2-low** | **HER2-low to HER2-zero** | **HER2-zero to HER2 positive** | **HER2-low to HER2 positive** | **Kappa (95% CI)** |
| **Bone metastasis**  **n=36** | 16 (44.4%) | 20 (55.6%) | 6 (16.7%) | 8 (22.2%) | 2 (5.6%) | 4 (11.1%) | 0.022  (-0.230 – 0.273) |
| **Liver**  **n=44** | 26 (59.1%) | 18 (40.9%) | 13 (29.5%) | 3  (6.8%) | 0 | 2 (4.5%) | 0.048  (-0.195 – 0.291) |
| **Lung / Pleura**  **n=6** | 6  (100.0%) | 0 | 0 | 0 | 0 | 0 | 1.0  (1.0 -1.0) |
| **Skin / Soft tissue**  **n=16** | 9  (56.3%) | 7 (43.7%) | 4 (25.0%) | 2  (12.5%) | 0 | 1 (6.3%) | 0.082  (-0.363 – 0.527) |
| **Lymph node**  **n=4** | 2  (50.0%) | 2 (50.0%) | 2 (50.0%) | 0 | 0 | 0 | 0.000  (0.000 – 0.000) |
| **CNS**  **n=10** | 2  (20.0%) | 8 (80.0%) | 5 (50.0%) | 1  (10.0%) | 0 | 2 (20.0%) | -0.250  (-0.606 – 0.106) |
| **Others**  **n=11** | 3  (27.3%) | 8 (72.2%) | 4 (36.4%) | 4 (36.4%) | 0 | 0 | -0.467  (-0.980 – 0.047) |
