# Supplementary material for: Discordance of HER2-Low between Primary Tumors and Matched Distant Metastases in Breast Cancer
Source: Cancers (Basel). 2023 Feb 23;15(5):1413. doi: 10.3390/cancers15051413 (PMC10000561; doi:10.3390/cancers15051413)
Supplement: Supplementary file 1 [file cancers-15-01413-s001.zip › Supplement/Table S4.docx]

**Table S4:** Change of HER2 status in different metastatic sites (n=148)

|  | **No change** | **HER2 change** | | | | |  |
| --- | --- | --- | --- | --- | --- | --- | --- |
|  |  | **Total** | **HER2-zero to HER2-low** | **HER2-low to HER2-zero** | **HER2-zero to HER2 positive** | **HER2-low to HER2 positive** | **Kappa (95% CI)** |
| **Bone metastasis**  **n=38** | 18 (47.4%) | 20 (52.6%) | 6 (15.8%) | 8 (21.1%) | 2 (5.3%) | 4 (10.5%) | 0.124  (-0.134 - 0.383) |
| **Liver**  **n=50** | 32 (64.0%) | 18 (36.0%) | 13 (26.0%) | 3  (6.0%) | 0 | 2 (4.0%) | 0.332  (0.095 – 0.570) |
| **Lung / Pleura**  **n=9** | 8  (88.9%) | 1 (11.1%) | 0 | 0 | 0 | 0 | 0.795  (0.418 – 1.0) |
| **Skin / Soft tissue**  **n=18** | 11  (6.1%) | 7 (38.9%) | 4 (22.2%) | 2  (11.1%) | 0 | 1 (5.6%) | 0.315  (-0.095 – 0.725) |
| **Lymph node**  **n=5** | 3  (60.0%) | 2 (40.0%) | 2 (40.0%) | 0 | 0 | 0 | 0.375  (-0.137 – 0.887) |
| **CNS**  **n=15** | 7  (46.7%) | 8 (53.3%) | 5 (33.3%) | 1  (6.7%) | 0 | 2 (13.3%) | 0.221  (-0.099 – 0.540) |
| **Others**  **n=13** | 5  (38.5%) | 8 (61.5%) | 4 (30.8%) | 4 (30.8%) | 0 | 0 | 0.000  (-0.538 – 0.538) |
