# Supplementary material for: Discordance of HER2-Low between Primary Tumors and Matched Distant Metastases in Breast Cancer
Source: Cancers (Basel). 2023 Feb 23;15(5):1413. doi: 10.3390/cancers15051413 (PMC10000561; doi:10.3390/cancers15051413)
Supplement: Supplementary file 1 [file cancers-15-01413-s001.zip › Supplement/Table S5.docx]

**Table S5:** Change of HER-2 in different molecular subtypes (luminal-like and triple-negative) (n=127)

|  | **No change** | **HER2 change** | | | | |  |
| --- | --- | --- | --- | --- | --- | --- | --- |
|  |  | **Total** | **HER2-zero to HER2-low** | **HER2-low to HER2-zero** | **HER2-zero to HER2 positive** | **HER2-low to HER2 positive** | **Kappa (95% CI)** |
| **Luminal A/B**  **n= 107** | 54 (50.5%) | 53 (49.5%) | 28 (26.2%) | 16 (15.0%) | 2 (1.9%) | 7 (6.5%) | -0.044  (-0.202 – 0.114) |
| **Triple-negative**  **n= 20** | 10 (50.0%) | 10 (50.0%) | 6 (30.0%) | 2 (10.0%) | 0 | 2 (10.0%) | 0.107  (-0.247 – 0.461) |
