# Supplementary material for: Discordance of HER2-Low between Primary Tumors and Matched Distant Metastases in Breast Cancer
Source: Cancers (Basel). 2023 Feb 23;15(5):1413. doi: 10.3390/cancers15051413 (PMC10000561; doi:10.3390/cancers15051413)
Supplement: Supplementary file 1 [file cancers-15-01413-s001.zip › Supplement/Table S6.docx]

**Table S6:** Change of HER2 status between primary tumor and metastasis in the de-novo cohort (n=53)

|  |  | **Metastasis** | | |
| --- | --- | --- | --- | --- |
| **Primary tumor** |  | **HER2-zero**  **(n=9, 17.0%)** | **HER2-low**  **(n=29, 54.7%)** | **HER2 positive**  **(n=15, 28.3%)** |
|  | **HER2-zero**  **(n=8, 15.1%)** | 2 (3.8%) | 6 (11.3%) | 0 |
|  | **HER2-low**  **(n=31, 58.5%)** | 7 (13.2%) | 22 (41.5%) | 2 (3.8%) |
|  | **HER2 positive**  **(n=14, 26.4%)** | 0 | 1 (1.9%) | 13 (24.5%) |
