# Supplementary material for: Discordance of HER2-Low between Primary Tumors and Matched Distant Metastases in Breast Cancer
Source: Cancers (Basel). 2023 Feb 23;15(5):1413. doi: 10.3390/cancers15051413 (PMC10000561; doi:10.3390/cancers15051413)
Supplement: Supplementary file 1 [file cancers-15-01413-s001.zip › Supplement/Table S7.docx]

**Table S7:** Change of HER2 status between primary tumor and metastasis in the secondary metastatic breast cancer cohort (n=95)

|  |  | **Metastasis** | | |
| --- | --- | --- | --- | --- |
| **Primary tumor** |  | **HER2-zero**  **(n=22, 23.2%)** | **HER2-low**  **(n=57, 60.0%)** | **HER2 positive**  **(n=16, 16.8%)** |
|  | **HER2-zero**  **(n=41, 43.2%)** | 11 (11.6%) | 28 (29.5%) | 2 (2.1%) |
|  | **HER2-low**  **(n=47, 49.5%)** | 11 (11.6%) | 29 (30.5%) | 7 (7.4%) |
|  | **HER2 positive**  **(n=7, 7.4%)** | 0 | 0 | 7 (7.4%) |
