# Supplementary figures and images for: Discordance of HER2-Low between Primary Tumors and Matched Distant Metastases in Breast Cancer
Source: Cancers (Basel). 2023 Feb 23;15(5):1413. doi: 10.3390/cancers15051413 (PMC10000561; doi:10.3390/cancers15051413)

**Figure S1:** Change of HER2 status between primary tumor and metastasis in the entire cohort (n=148)
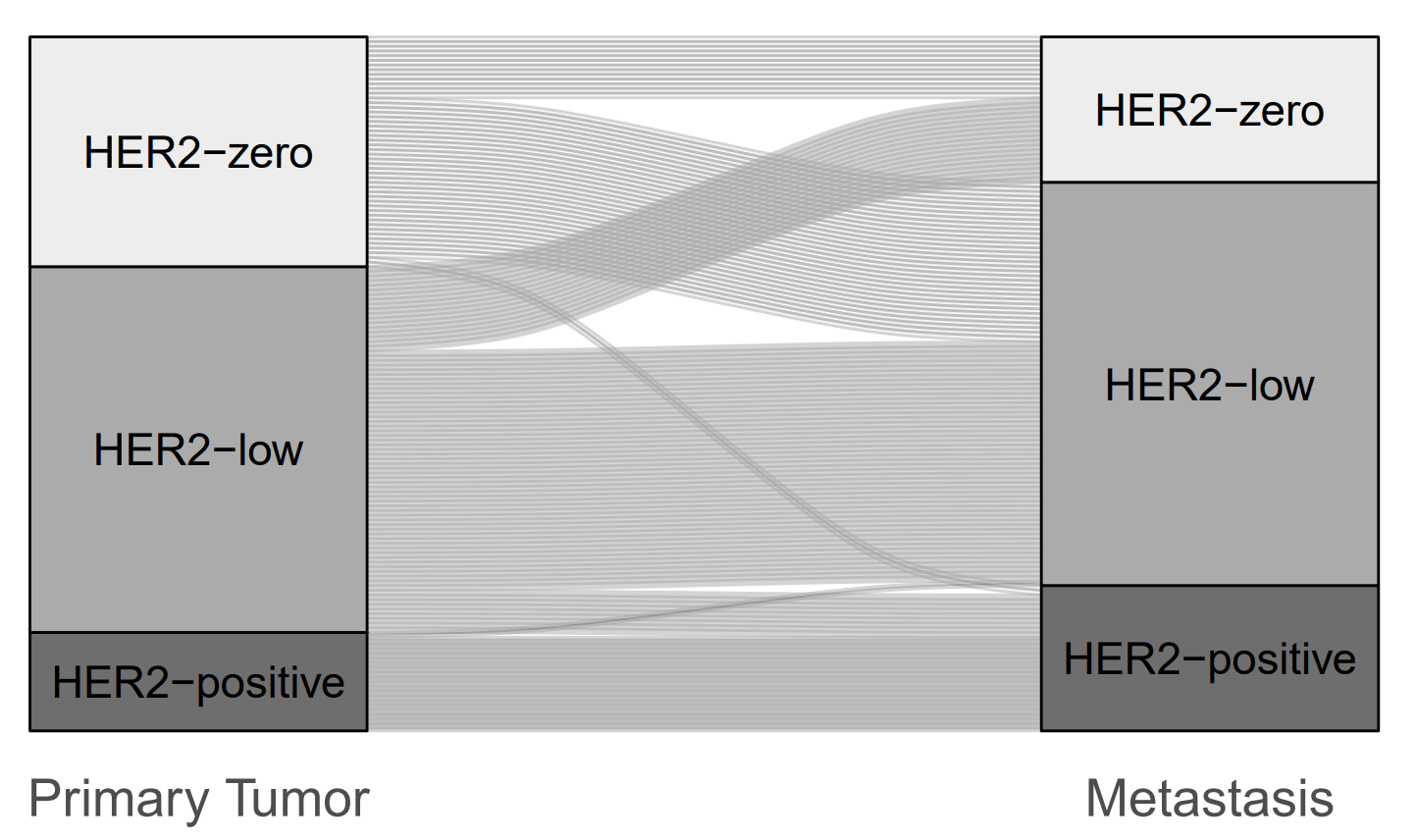

Supplement: Supplementary file 1 [file cancers-15-01413-s001.zip › Supplement/Figure S1.docx]

**Figure S2:** Change of HER2 status in different metastatic sites (n=148)


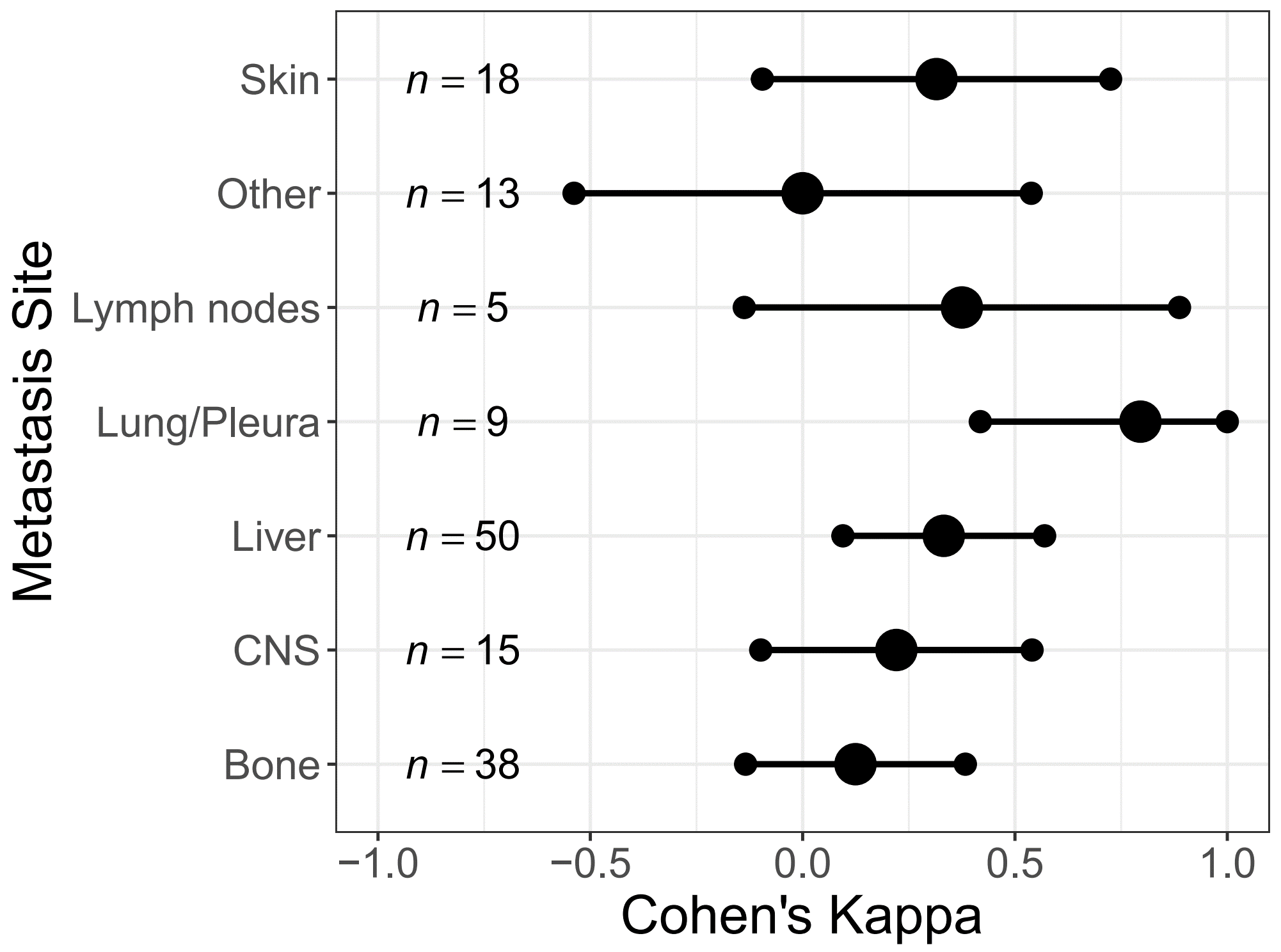

Supplement: Supplementary file 1 [file cancers-15-01413-s001.zip › Supplement/Figure S2.docx]

**Figure S3:** Change of HER-2 in different molecular subtypes (luminal-like and triple-negative) (n=127)


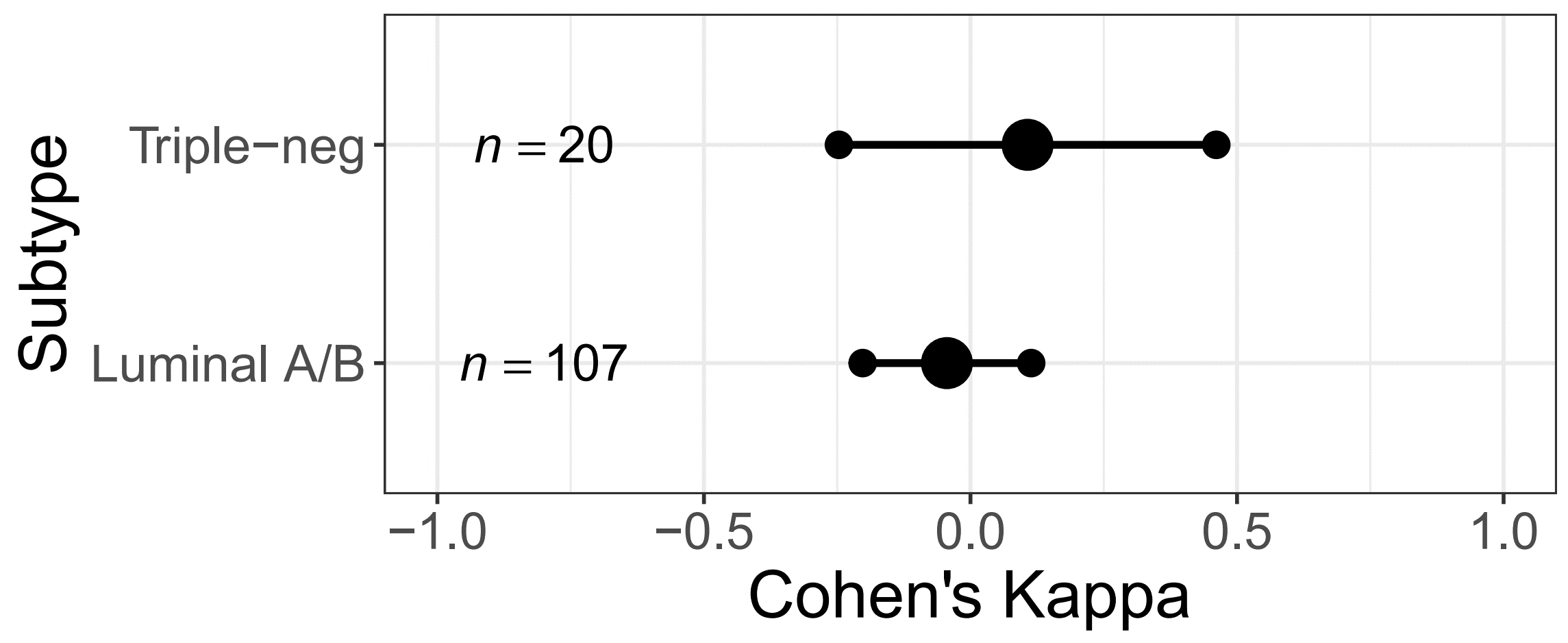

Supplement: Supplementary file 1 [file cancers-15-01413-s001.zip › Supplement/Figure S3.docx]
